# Co-Infection and Wild Animal Health: Effects of Trypanosomatids and Gastrointestinal Parasites on Coatis of the Brazilian Pantanal

**DOI:** 10.1371/journal.pone.0143997

**Published:** 2015-12-14

**Authors:** Natalie Olifiers, Ana Maria Jansen, Heitor Miraglia Herrera, Rita de Cassia Bianchi, Paulo Sergio D’Andrea, Guilherme de Miranda Mourão, Matthew Edzart Gompper

**Affiliations:** 1 Laboratório de Biologia e Parasitologia de Mamíferos Reservatórios, Instituto Oswaldo Cruz, Fundação Oswaldo Cruz, Rio de Janeiro, Rio de Janeiro, Brazil; 2 Laboratório de Biologia de Tripanosomatídeos, Instituto Oswaldo Cruz, Fundação Oswaldo Cruz, Rio de Janeiro, Rio de Janeiro, Brazil; 3 Laboratório de Parasitologia Animal, Universidade Católica Dom Bosco, Campo Grande, Mato Grosso do Sul, Brazil; 4 Departamento de Biologia Aplicada à Agropecuária, Universidade Estadual Paulista “Júlio de Mesquita Filho”, Jaboticabal, São Paulo, Brazil; 5 Laboratório de Vida Selvagem, Centro de Pesquisa Agropecuária do Pantanal, Empresa Brasileira de Pesquisa Agropecuária, Mato Grosso do Sul, Corumbá, Brazil; 6 Department of Fisheries and Wildlife Sciences, University of Missouri, Columbia, Missouri, Unites States of America; Universidad Nacional Autonoma de Mexico, MEXICO

## Abstract

Wild animals are infected by diverse parasites, but how they influence host health is poorly understood. We examined the relationship of trypanosomatids and gastrointestinal parasites with health of wild brown-nosed coatis (*Nasua nasua*) from the Brazilian Pantanal. We used coati body condition and hematological parameters as response variables in linear models that were compared using an information theoretic approach. Predictors were high/low parasitemias by *Trypanosoma cruzi* and *T*. *evansi*, and indices representing the abundance of distinct groups of gastrointestinal parasites. We also analyzed how host health changed with host sex and reproductive seasonality. Hemoparasites was best related to coati body condition and hematological indices, whereas abundance of gastrointestinal parasites was relatively less associated with coati health. Additionally, some associations were best predicted by models that incorporated reproductive seasonality and host sex. Overall, we observed a lower health condition during the breeding season, when coatis are under reproductive stress and may be less able to handle infection. In addition, females seem to handle infection better than males. Body condition was lower in coatis with high parasitemias of *T*. *evansi*, especially during the reproductive season. Total red blood cell counts, packed cell volume, platelets and eosinophils were also lower in animals with high *T*. *evansi* parasitemias. Total white blood cell counts and mature neutrophils were lower in animals with high parasitemias for both *Trypanosoma* species, with neutrophils decreasing mainly during the reproductive season. Overall, decreases in hematological parameters of females with *T*. *evansi* high parasitemias were less evident. For *T*. *cruzi*, monocytes decreased in individuals with high parasitemias. High abundances of microfilariae in the bloodstream, and cestode eggs and coccidian oocysts in feces were also associated with coati blood parameters. This study shows the potential value of examining hematological parameters as an approach to better understand the ecological relevance of parasite-host interactions.

## Introduction

Through their influence on host health, parasites may negatively affect several aspects of the life history of their hosts, including fecundity and survival [1–3]. As secondary effects, host abundance may decline and alter community or ecosystem structure and function [4]. Evaluating host health is therefore critical for understanding how parasites influence hosts at the individual and population levels.

Studies on the impact of parasites on the health of wild, free-living animals are usually limited to correlations between body condition indexes and parasite presence or intensity [5–9]. Alternatively, fluctuations in hematological parameters may be used to predict fitness [10,11] and have been used as indicators of the health of individual animals in veterinary medicine for decades [12]. Red blood cells distribute oxygen throughout the body and their common laboratory measurements (packed cell volume, total red blood cell count and hemoglobin concentration) are indicative of anemia when below normal values [12,13]. Peripheral neutrophils are indicators of acute inflammatory response, as their numbers increase rapidly due to cytokines released during tissue injury; an increase in the number of circulating young neutrophils (called band cells or band neutrophils) also occurs when demand from the bone marrow increases and a large percentage of these young cells are released in the bloodstream [12]. Lymphocytes are the effectors of acquired immunity and proliferate in response to antigenic stimuli; their circulating levels therefore may be useful indicators of current immunological investment [10,12]. Peripheral monocyte numbers, in turn, increase due to subacute and chronic inflammatory responses, usually caused by bacterial and protozoan infections [12]. The role of eosinophils is manifold and still not completely elucidated, but they are involved in allergic and inflammatory responses; eosinophilia (increased number of circulating eosinophils) can be found as a response to several parasitic infections [14–17]. Platelets are multifunctional, playing role for instance in haemostasis and thrombosis, vessel repair, inflammation and host defense including immunity to parasites [18,19].

Host sex and season can mediate how parasite burdens ultimately influence host health. Seasonal changes in host behavior and contact rates, for instance, may influence host-parasite interactions and host immune defenses [20]. Nutritional depletion associated with the dry season in some areas can also compromise individual health [21]; moreover, if mating is seasonal, the immunosuppression usually associated with an increase in sex hormones and the higher costs of reproduction can be added to those of climate, thereby promoting a stress response that negatively affects host health [21,22]. Additionally, in mammals and some other *taxa*, males may be more heavily parasitized than females due to sex-specific behavior, diet, life-history particularities, as well as androgen levels, physiological stress, and/or resource allocation tradeoffs with immune function [23–29].

Brown-nosed coatis are among the most common South American carnivores; they have a complex social structure in which adult females and immature individuals form groups of up to 30 individuals [30], while most adult males are solitary and attempt to join groups only during the reproductive season [31]. Coatis harbor parasites that infect other wildlife, domestic animals, and humans [32–36], and the impacts of some of these species on coati health have been initially investigated with an emphasis given to *Trypanosoma cruzi* and *T*. *evansi* infection under natural and experimental conditions [37–40]. These hematozoans are of public health and economic importance since they cause Chagas disease in humans and “Mal de Cadeiras” disease in horses, respectively, and coatis have been suggested as reservoir hosts for both species [41–44]. Both *T*. *cruzi* and *T*. *evansi* are primarily transmitted by arthropod vectors, but while *T*. *cruzi* transmission depends on the development of the parasite in the intermediate host (kissing bugs, Hemiptera: Reduviidae), *T*. *evansi* is transmitted mechanically through the mouthparts of one of several species of blood-sucking flies (Diptera).

*Trypanosoma evansi* was observed to cause anemia in coatis [37,39,45] whereas *T*. *cruzi* was previously associated to lower total leukocyte counts [40]. Other parasites may also influence coati health. For example, some species of filarid nematodes, such as *Dirofilaria immitis*, can cause loss of body condition and anemia [46], although filarid parasites from coatis are poorly known and their effect on host health has not been investigated. Strongylids (*e*.*g*. hookworms) ingest blood and may can cause anemia and loss of body condition [47] so that high intensities of these parasites are expected to alter the hematological profile of coatis. Other gastrointestinal parasites such as cestodes, acanthocephalans and coccidians may also influence coati body condition. Cestodes and acanthocephalans, for instance, sequester nutrients before absorption by the host intestinal tract and may decrease host condition when in high intensities. Finally, concomitant infection by multiple species of parasites is common and may worsen host health condition [48,49].

By collectively examining how these hemoparasites and helminths influence coati health in natural settings, we can gain an understanding of how parasite communities influence broader populations through their effects on individual animals. In this study, we examined the importance of two species of trypanosomatids (*Trypanosoma cruzi* and *T*. *evansi*) and 5 groups of additional parasites (filarids, cestodes, nematodes, acanthocephalans and coccidians) on the health of free-ranging brown-nosed coatis (Procyonidae: *Nasua nasua*) from the Brazilian Pantanal as a model for investigating the effects of concomitant infection by micro and macroparasites on the host health. In addition, we analyzed how health condition changes according to host sex and reproductive seasonality (reproductive vs. non-reproductive season); we found that hemoparasites (tripanosomatids and microfilariae) best predicted coati body condition and hematological indices, with some associations best predicted by models that also incorporated reproductive seasonality and host sex. The potential value of examining hematological parameters as an approach to better understand the ecological relevance of parasite-host interactions was confirmed.

## Materials and Methods

### Study area

Field work was conducted at Nhumirim Ranch (18°59’S, 56°39’W), a 4400 ha research station of The Brazilian Agricultural Research Corporation (Embrapa) located in the Pantanal wetlands. Pantanal is characterized by sandy soil with a mosaic vegetation of semi-deciduous forest, dispersed shrub vegetation, and seasonally flooded fields. Human population density is low (< 2 people per km^2^) and the main economic activity is cattle ranching [50]. The climate is tropical, with two marked seasons: a wet season (October to March) and a dry season (April to September). In the Nhumirim Ranch, the reproductive season of coatis starts by early August and extends to late November [51].

### Capture procedures

From May 2006 to February 2009 we captured coatis up to four times per year, using wire box traps (1m x 0.40m x 0.50m) placed in a trapping grid of 7.2 Km^2^ and with occasional traps placed outside of the grid. Traps were baited with bacon, set late in the afternoon and checked in the following morning. We anesthetized individuals with an intramuscular injection of Zoletil^@^50 (Virbac^®^; tiletamine hydrochloride and zolazepan hydrochloride, 10 mg/Kg), ear-tagged them with numbered colored tags (Nasco Rototags^®^) and marked them with subcutaneous transponders (AnimalTag^®^). Body size measures, body mass, as well as tooth eruption and wear were recorded and used to age individuals [52]. Animals were released at the capture site.

We collected and stored blood samples (up to 5mL) in Vacutainer^®^ tubes with EDTA. In addition, we prepared eight blood smears from captured animals and collected fecal samples from beneath traps or via fecal loop. Handling and sample collection procedures were standardized to minimize biases in the hematological profiles caused by an alarm response due to capture stress [53].

### Ethics Statement

Field permit to capture coatis in the study area and to sample collection was granted by the Brazilian Government Institute for Wildlife and Natural Resources Care (IBAMA, first license #183/2005 –CGFAU/LIC; last license #11772–2). Procedures adopted in this study were approved by the University of Missouri Animal Care and Use Committee (protocol #4459) and are in accordance with the recommendations in the Guidelines of the American Society of Mammalogists for the use of wild mammals in research [54]. All animals were monitored until recovery from anesthesia and all efforts were made to minimize stress. Appropriate biosafety techniques and individual protection equipment were used during animal handling and sample manipulation.

### Hematology

Packed cell volume (PCV), total red blood cell (RBC) counts and total white blood cell (WBC) counts were quantified within 8 hours of blood collection [13]. A subsample of whole blood was diluted 1:200 in distilled water and then placed in a Neubauer chamber where RBCs were immediately counted; total WBC counts were done in the Neubauer chamber after diluting whole blood at 1:20 in a 4% acid acetic solution for 10 minutes [13].

Blood smears were air-dried and fixed with methanol (Merk^®^). Slides were stained with Giemsa’s azure eosin methylene blue solution (Merk^®^) for differential leukocyte counts (that is, count of band neutrophils, mature neutrophils, eosinophils, lymphocytes, monocytes and basophils) and observation of blood cell morphology [13]. We diluted 10 mL Giemsa’s azure eosin methylene blue solution with 190 mL buffer solution (pH 6.8–7.0) and stained the smears for 15−20 minutes. We counted and classified 100 leukocytes across the entire cell monolayer using the crossing technique [13]. For animals showing leukocytosis (>25,000 WBC/ mm^3^), we counted 200 leukocytes to improve the accuracy of estimates. In addition, we characterized RBC morphology (cell shape and color), and recorded the number of metarubricytes (young, nucleated red blood cells). Red blood cells in the individual blood smears were characterized as altered or normal. They were qualified as altered when polycromasia (cells with distinct colors) and poikilocytosis (distinct sizes) were predominant or when more than 3 metarubricytes/100 leukocytes were found in blood smears. We also recorded the number of platelets (averaged number in 10 microscope fields) found during leukocyte counts. Blood smears for which we observed platelet aggregation were not included in the analysis as platelet aggregation may be an artifact of smear preparation and can influence total platelet count.

### Parasite identification and quantification

We used the microhematocrit centrifuge technique (MHCT) [55] with blood subsamples from the captured animals to assess high parasitemia infections by *T*. *evansi* [40] and microfilariae; and hemoculture (HC) to assess high parasitemia by *T*. *cruzi*. Microfilariae are a blood-borne stage in the life cycle of filarid worms. We estimated the average number of microfilariae in 10 randomly chosen 100× microscope fields during MHCT.

Hemoculture (HC) was used to assay for epimastigote forms of *T*. *cruzi* and for isolating them for subsequent molecular characterization, as described elsewhere [40]. *Trypanosoma evansi* was directly identified in the stained blood smears after detection of trypomastigote forms with the MHCT technique. *Trypanosoma cruzi* and *T*. *evansi* can be distinguished on Giemsa-stained smears, as *T*. *evansi* does not show a visible kinetoplast while *T*. *cruzi* has a terminal/subterminal kinetoplast and shows a characteristic “C-shape”. Moreover, *T*. *evansi* appear in high numbers in the MHCT but does not grow in HC. Positive HC and/or MHCT were used as indicators of high parasitemias and infectivity competence [43], since detection of trypomastigote forms in the bloodstream means the vectors have relatively higher chances of becoming infected (and therefore potentially transmitting) the parasite when taking a blood meal; negative HC/MHCT, in turn, are equivalent to low/no parasitemia [40]

Beginning in August 2007, we quantified eggs of helminths and oocysts of gastrointestinal protozoans found in fecal samples as the number of eggs/oocyst per gram of feces. A subsample of 1−3 g of feces was weighed to the nearest 0.01 g, suspended in 20 mL of 10% formalin and filtered through a gauze mesh. The diluted sediment was then divided in two 15mL tubes, one of which was analyzed by sugar solution flotation (sugar density 1.27) and sedimentation techniques [56]. After sedimentation, the pellet was suspended with 1mL of 10% formalin and a subsample of 80 μL from this solution was placed on a slide for analysis [57]. Slides from the sugar flotation and sedimentation techniques were analyzed at 100× and all egg and oocyst types were photographed under 400×. Eggs and oocyst were measured and classified into morphotypes. The total number of each egg and oocyst found with the sedimentation technique was extrapolated to the total weight of feces present in the 15 mL tube (usually 1.5 grams). To this total, we added the eggs found during the sugar flotation done with the same material.

Identification at the species level was not possible due to lack of information on helminths of coatis. We therefore used an aggregate-scale approach [11], that is, we identified and grouped eggs/oocysts at the most specific *taxa* possible. Therefore, in addition to the filarids (microfilariae) quantified with the MHCT technique, we considered four groups of gastrointestinal parasites in the aggregate-scale analyses: Eimeriidae spp (coccidians), Oligacanthorhynchidae sp. (acanthocephalans), Strongylida spp. (hookworms) and Cestoda spp. Other groups had low prevalences and were not analyzed. Aggregate-scale analyses as well as the egg/oocyst and filarid quantifications employed are not refined methods to quantify parasite abundance due to factors such as sampling heterogeneity and resource competition between parasitic species leading to density-dependent suppression of egg production [58,59]. However, physiological effects, if strong, can nonetheless be detected using such aggregate-scale approaches [11].

### Statistical analyses

There is no clear consensus regarding the most suitable condition index [60,61]. We opted to assess variation in coati body condition based on the standardized residuals from an ordinary linear regression between body mass (g) and head-body length (mm) of individuals, while accounting for age and sex effects. This should circumvent the effects of animal growth on the condition index. Therefore, the residuals were calculated for males and females separately because coatis are dimorphic in size and after residual calculations, only greater than 2 years old coatis were considered. We excluded pregnant/lactating females from the analyses, as we know this condition affect both their hematological profile and body condition, regardless of their health condition and the presence of parasites. Seasonality was broadly categorized as reproductive (August to November) or non-reproductive (January to early July). We combined Log_10_RBC and PCV values on subsequent analyses by using the factor scores of the first component of a principal component analysis (PCA), since those variables are highly correlated. Total WBC and leukocyte differential numbers were log_e_-transformed for normalization prior to analyses, except for band cells, which showed a negative binomial distribution and were analyzed accordingly; and platelet numbers, which were squared root transformed before analysis. The RBC morphology was compared using one-tailed Fisher’s Exact tests between categories of qualitative variables (host sex, season and *T*. *cruzi* and *T*. *evansi* high parasitemias). In these analyses, recaptures were not considered to avoid pseudo-replication (see below).

We used an information theoretic approach to contrast the relative performance of models that predict measures of host health parameters. We created linear models accounting for seasonal dynamics (reproductive vs. non-reproductive season), host sex, and parasite abundance to investigate the relative importance of these factors for body condition and hematological parameters of individual coatis. The standardized residuals of body size (body condition), the PCA factor representing PCV and RBC, total WBCs and each leukocyte type count, as well as platelet numbers were the response variables in the models. Basophils were not analyzed because they were relatively less encountered. The number of band cells was modeled using generalized linear models with a negative binomial distribution and log link. Data from recaptured individuals (obtained about every 3 months) were eliminated from data modeling to avoid pseudo-replication, but were included in mean/median and 95% confidence interval estimations. For seven recaptured animals showing low and high *T*. *cruzi* parasitemias in different field excursions, we compared the numbers of distinct blood cells between periods of low and high parasitemias using paired t-test. A similar approach was not performed for *T*. *evansi* because of sample size limitations.

Because combining all independent variables in a single set of models would result in an overly complex analysis with many models and a reduced sample size of egg/oocyst counts, which were initiated in 2007, we ran two separate sets of analyses. Sample sizes based on blood collection were larger than fecal samples, so we first contrasted a set of models with *T*. *cruzi*, *T*. *evansi*, microfilariae (hemoparasites) and extrinsic variables (host sex and seasonality) and then proceeded by adding each gastrointestinal parasite to the best-fitting model of hemoparasites and extrinsic variables. In this set of models, high parasitemias by *T*. *cruzi* and *T*. *evansi*, host sex, and seasonality were qualitative descriptors and average microfilariae/field was a quantitative variable. We created model sets including all possible additive combinations of independent predictors of hemoparasites and extrinsic variables (32 models in total); furthermore, interaction terms were included in additional models whenever investigation of predictor vs. response variable plots revealed possible interactions between these variables.

Candidate models represented independent hypotheses, and model fit was compared using Akaike Information Criterion corrected for small sample size (AICc) and for overdispersion when needed (QAICc). Models were ranked based on the difference between the best approximating model (model with the lowest AICc or QAICc) and all others in the set of candidate models (ΔAICc, ΔQAICc). Models with differences within two units of the top model were considered to have strong empirical support and were considered competitive models [62]. However, larger models that differ from the best fitting model by one parameter and had essentially the same maximized log-likelihood as the best model were not considered to be competitive; when there was no clear best-fitting model, the relative importance of each predictor was quantified by adding the Akaike weights across all the models that included that predictor (variable weight) [62]. Variables weights lower than 0.40 were not discussed due to the relatively low variable importance. In addition, variable weights for interaction terms were not calculated because those were included in a relatively small proportion of models [62]. When the best-fitting model was the intercept-only model, we did not proceed with model analysis and interpretation.

Since sample size for gastrointestinal parasites was small, we did not run models with all possible combination of gastrointestinal parasites, hemoparasites and extrinsic variables. Instead, we added each gastrointestinal parasite predictor to the best-fitting hemoparasite and extrinsic variables model (see above) and observed whether the model fit was improved. In this way, we accessed the relative importance of hemoparasites, gastrointestinal parasites and extrinsic variables on coati health. The global model containing all gastrointestinal parasites was included in the set of models in order to calculate c-hat and verify the need of using QAICc rather than AICc for model comparison. A total of 63 fecal samples were analyzed for gastrointestinal parasites, but sample size used to run the models varied from 16 (for models in which platelet numbers were the dependent variable) to 31 (for models of most hematological variables); this is so because a) we considered only one capture for each coati individual in the model analyses and b) not all samples analyzed for gastrointestinal parasites had the other independent variables available.

## Results

One hundred and two blood samples from 70 adult coatis (22 females, 48 males) were analyzed for the presence of *Trypanosoma* spp., microfilariae and hematological parameters. Twenty-nine coatis showed high parasitemias of *T*. *cruzi* (positive HC) and 20 showed high parasitemias of *T*. *evansi* (positive MHCT) at least once during the study span. Three individuals (2 females and 1 male) showed concomitant high parasitemias by both *T*. *cruzi* and *T*. *evansi*. Microfilariae were found in 93% of blood samples and 91% of the individuals analyzed, with abundance varying from 0 to 17.9 microfilariae/microscope field. Twenty-eight samples from distinct animals were considered in the analysis for the presence of gastrointestinal parasites. The most common parasites were coccidians (14 infected coatis), followed by acantocephalans (7), cestodes (7) and strongylids (5).

Body condition was best related to season, *T*. *evansi* parasitemia, and with less magnitude to abundance of microfilariae (Tables 1 and 2). Body condition was lower during the reproductive season (β = - 0.64) and with positive MHCT by *T*. *evansi* (β = - 0.56) and microfilariae abundance (β = - 0.06). On average, coatis with high parasitemias by *T*. *evansi* were 350 grams lighter (nonstandardized residuals) than negative MHCT coatis. This represents a 6 to 10% lower total body mass of males and females, respectively. The average residuals were approximately 800 grams lower for animals with high parasitemias during the reproductive season (15 to 23% lower body mass on males and females, respectively). *Trypanosoma cruzi* parasitemias and host sex had a relationship not worthy of notice on body condition (var. weight = 0.28; Table 2).

**Table 1:** Best-fitting models describing body condition and hematological parameters of coatis.

Model	Log(l); log(l)/c	AICc; QAICc	k	ΔAICc; ΔQAICc	AICc weight; QAICc weight
*Body condition*					
Season + TE + Mf	-90.9	192.7	5	0.00	0.22
Season + TE + TC + Mf	-90.3	194.0	6	1.35	0.11
Season + TE	-92.7	194.1	4	1.38	0.11
Season + TE + Mf + Sex	-90.53	194.41	6	1.73	0.09
*RBC & PCV*					
TE + Mf	-93.4	195.4	4	0.00	0.18
Mf + TC + TE	-92.4	195.8	5	0.36	0.15
Sex + Mf + TE	-93.0	196.9	5	1.48	0.08
TE	-95.4	197.1	3	1.67	0.08
Sex + Mf + Season × TE	-90.8	197.4	7	1.94	0.07
*WBC*					
Sex × TE + TC	-31.0	75.3	6	0.00	0.49
*Mature neutrophils*					
Season × TC + Season × TE + Sex × TE	-38.9	98.8	9	0.00	0.34
TC × Season + Sex × TE x Season	-36.4	99.4	11	0.63	0.25
Season × TC + Season × TE + Sex × TE + Sex × TC	-38.3	100.3	10	1.51	0.16
Season × TC + Sex × TC + Season × Sex × TE	-35.6	100.7	12	1.93	0.13
*Band neutrophils*					
Season + Sex × TE + Mf	-199.8	415.3	7	0.00	0.29
Season + Sex × TE + Mf + TC	-199.0	416.4	8	1.07	0.17
*Eosinophils*					
Season + TE	-82.8	174.2	4	0.00	0.26
Season + TE + Mf	-82.1	175.2	5	0.92	0.17
Season + TE + Sex	-82.3	175.6	5	1.32	0.14
*Platelets*					
TE	-37.5	84.5	4	0.00	0.28
Intercept	-39.2	85.3	3	0.77	0.19

Season = reproductive x non-reproductive seasons, Mf = microfilariae abundance; TE = *T*. *evansi* parasitemia; TC = *T*. *cruzi* parasitemia; Sex = host gender; RBC & PCV = PCA factor scores for red blood cell count (mm^3^) and packed cell volume (%); WBC = White blood cell count. Only models with ΔAICc (ΔQAICc) ≤ 2 are shown. Results are not shown when the intercept-only was the best fitting model.

**Table 2:** Variable weight for the best-fitting models describing body condition and hematological parameters of coatis.

Variables	Variable weight
*Body condition*	
Season	0.85
TE	0.74
Mf	0.63
TC	0.28
Sex	0.28
*RBC & PCV*	
TE	0.89
Mf	0.73
TC	0.38
Sex	0.32
Season	0.24
*Band cells*	
Season	0.96
Mf	0.85
TC	0.34
*Eosinophils*	
Season	0.95
TE	0.91
Mf	0.34
Sex	0.32

Variable weights for interaction terms are not shown. See Table 1 for variable abbreviations.

**Fig 1:**
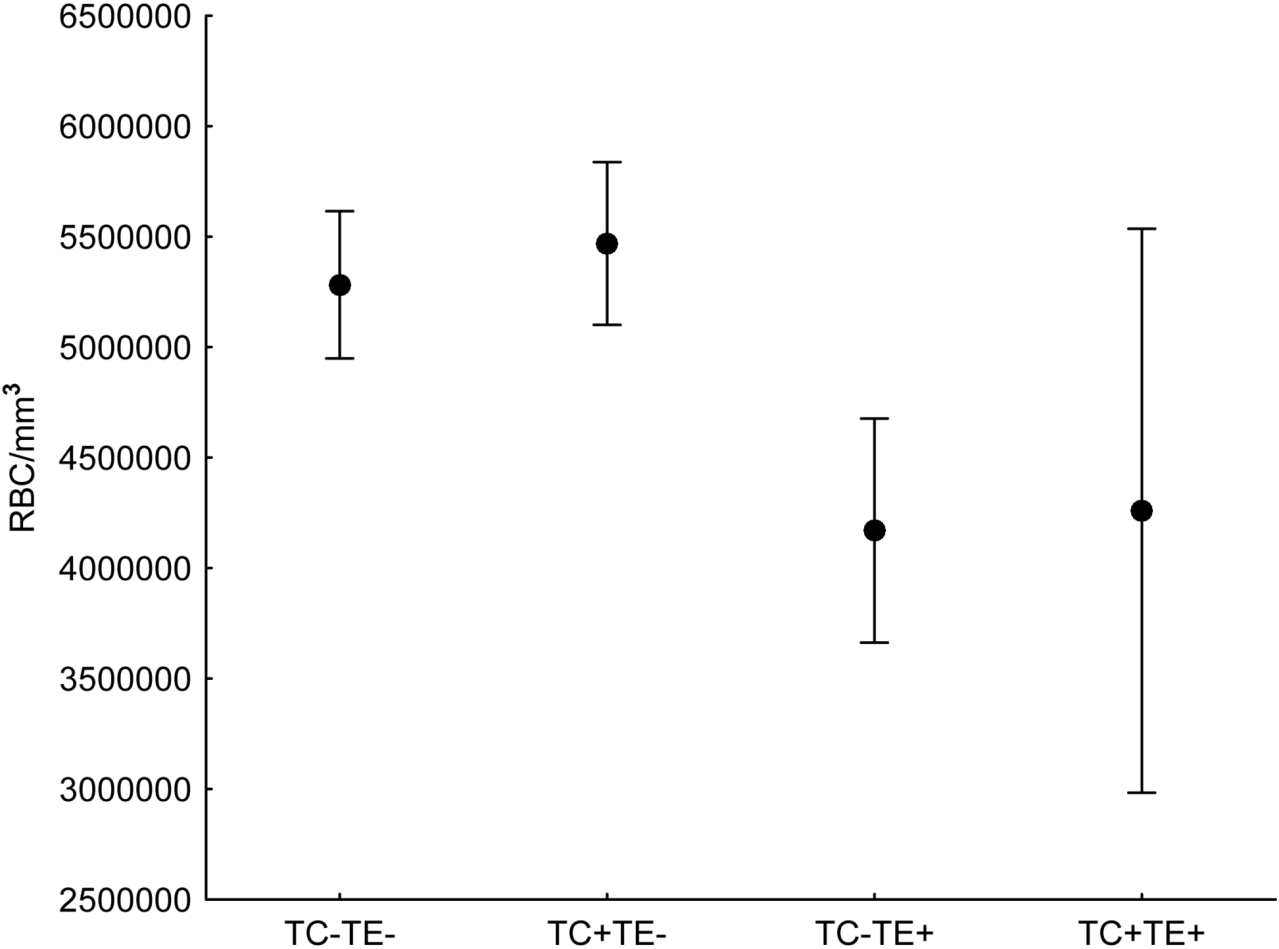
Red blood cells as a function of *Trypanosoma* parasitemia in coatis. Total red blood cells count (RBC) as a function of high (+) or low (-) parasitemias by *Trypanosoma evansi* (TE) and *T*. *cruzi* (TC) in coatis captured in the Nhumirim Ranch, Pantanal from 2005 to 2009. High parasitemia by *T*. *evansi* was detected by the microhematocrit centrifuge technique (MHCT), whereas for *T*. *cruzi* it was detected by hemoculture (HC). Bars are 95% confidence limits.

Total number of RBCs and PCV values was best predicted by *T*. *evansi* parasitemias (β = - 1.092) and microfilariae abundance (β = - 0.06). Although *T*. *cruzi* high parasitemias (positive HC) was in the second best-fitting model, its relative importance was small (var. weight = 0.38; Table 2); the contribution of *T*. *cruzi* parasitemia was mostly represented by the double-infected animals (TC+TE+), which showed an even lower mean PCV and RBCs, although confidence interval are wide for this category due to small sample size (N = 3; Fig 1). The lower RBC and PCV counts were accompanied by alterations in the shape and color of RBC, with 21% of coatis with high *T*. *evansi* parasitemias showing altered RBCs (P < 0.005). In contrast, individuals with positive HC by *T*. *cruzi* did not show alterations on RBC (P = 0.630). There were no differences between sexes (P = 0.301) or seasons (P = 0.593) in the morphology of RBCs.

**Fig 2:**
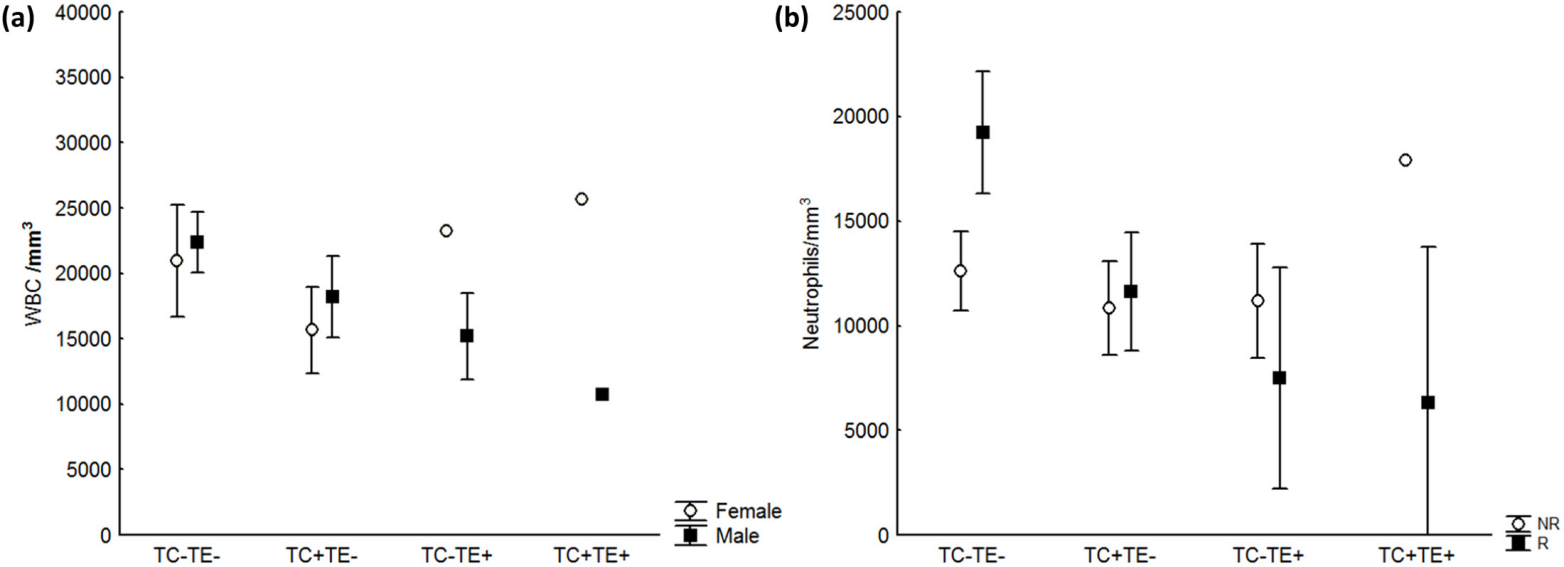
White blood cells and neutrophils as a function of *Trypanosoma* parasitemia in coatis. (a) Total white blood cells count (WBC) as a function of high (+) or low (-) parasitemias by *Trypanosoma evansi* (TE) and *T*. *cruzi* (TC) in coati males and females and (b) number of mature neutrophils as a function of *Trypanosoma* species in coatis during the reproductive (R) and non-reproductive seasons (NR). The study was conducted in the Nhumirim Ranch, Pantanal from 2005 to 2009. High parasitemia by *T*. *evansi* was detected by the microhematocrit centrifuge technique (MHCT), whereas for *T*. *cruzi* it was detected by hemoculture (HC). Bars are 95% confidence limits.

Total WBC count was best predicted by both *Trypanosoma* species (Table 1), with a lower WBC count that was particularly evident among males with high parasitemias by *T*. *evansi* (Fig 2a). Females with high parasitemias by *T*. *evansi*, in turn, showed higher mean WBC, although confidence limits were wide due to small sample size of females with high parasitemias (N = 4; Fig 2a). The influence of *T*. *cruzi* and *T*. *evansi* on WBCs was mainly a consequence of changes in the number of mature neutrophils, which were lower in animals with high parasitemias by these two parasites, especially during the reproductive season (Table 1 and Fig 2). Such lower number was evident in the reproductive season because the overall number of mature neutrophils in animals with negative HC and MHCT (low parasitemias by both *Trypanosoma* species) was higher in that season (Fig 2b). As with the WBC results, males with high parasitemias by *T*. *evansi* also showed a lower mean ± CI number of mature neutrophils (8507 ± 2925 compared to 14163 ± 1575 cells/mm^3^ in negative MHCT males).

The number of band neutrophils was best predicted by season, abundance of microfilariae, sex, and *T*. *evansi* parasitemia (Tables 1 and 2). During the non-reproductive season, the number of band cells remained constant and similar in both sexes (median = 112.5/mm^3^), despite high parasitemias by *T*. *evansi* or *T*. *cruzi*. During the reproductive season, band cells were higher (median = 214/mm^3^), especially in *T*. *evansi* negative males (median = 674.5/mm^3^), but were lower in individuals with high parasitemias by *T*. *evansi* (median = 72.5 / mm^3^), whereas there was only one female with high *T*. *evansi* parasitemia during that season. In addition, band neutrophil numbers were lower when abundance of microfilariae was high (β = - 0.19). The relationship between *T*. *cruzi* parasitemia and the number of band cells may be considered of little importance (var. weight = 0.34; Table 2).

Circulating eosinophil numbers were lower in animals with high parasitemias by *T*. *evansi* (the median changed from 1389/mm^3^ to 606/mm^3^) and during the reproductive season (from 1308.5/mm^3^ to 1011/mm^3^). Even though lymphocytes are the effectors of acquired immunity and their circulating levels may be useful indicators of current immunological investment [12,53], none of the variables analyzed predicted variation on the total number of lymphocytes, for which the intercept-only model was the best-fitting model. The same was true for monocytes, although by comparing the hematological profile of the same individuals during high and low *T*. *cruzi* parasitemias, we detected a marginally significant decrease from 915 to 478 monocytes/mm^3^ (paired t-test = 2.41; d. f. = 6; P = 0.052), while other leukocyte numbers did not differ (P > 0.05). Finally, the number of platelets was lower in animals with high parasitemias by *T*. *evansi* (a median of 13.9 compared to 25.3 platelets/microscope field; Table 2).

In general, incorporation of data on gastrointestinal parasites did not result in a more supported model. An exception was in models predicting eosinophil and neutrophil numbers (Table 3). The abundance of cestode eggs was related to higher numbers of eosinophils (β = 0.002), whereas the abundance of coccidian oocysts (Eimeriidae spp.) was associated with higher numbers of neutrophils (β = 0.003).

**Table 3:** Ranking of general best-fitting models including gastrointestinal parasites and describing body condition and hematological parameters on coatis.

Model	Log(l); log(l)/c	AICc; QAICc	k	ΔAICc; ΔQAICc	AICc weight; QAICc weight
*Body condition*					
Season + TE + Mf	-37.3	87.4	6.0	0.0	0.29
Season + TE + Mf + Strongilyda	-36.0	88.0	7.0	0.7	0.20
Intercept	-36.1	88.3	7.0	0.9	0.18
Season + TE + Mf + Oligacanthorhynchidae	-42.1	88.7	3.0	1.3	0.15
*RBC & PCV*					
TE + Mf	-39.1	87.9	4.0	0.0	0.39
TE + Mf + Eimeriidae	-38.0	88.8	5.0	0.9	0.25
*Neutrophils*					
Season x TC + Season × TE + Sex × TE + Eimeriidae	-8.6	50.1	10.0	0.0	0.76
*Eosinophils*					
Season + TE + Cestoda	-26.8	66.6	5.0	0.0	0.53
Season + TE	-29.1	68.0	4.0	1.4	0.26

k = number of parameters in the models. See Table 1 for abbreviations. Results are not shown when the intercept-only was the best fitting model. Only models with ΔAICc (ΔQAICc) ≤ 2. Results are not shown when the intercept-only was the best fitting model. See Table 1 for variable abbreviations.

## Discussion

### Possible effects of *Trypanosoma evansi*

The detection of *T*. *evansi* presence was a predictor of lower numbers of RBCs and PCV (Table 1 and Fig 1), and of alterations in the overall morphology of RBCs and number of metarubricytes. This result confirms a preliminary investigation in which the impact of *T*. *cruzi* and *T*. *evansi* parasitemias on PCV values of the coatis was evaluated [40].

RBC and PCV values of coatis with high *T*. *evansi* parasitemias were similar to those found in other studies; this parasite causes a macrocytic normochromic anemia in coatis that with time may become normocytic normochromic, *i*.*e*., anemia without morphological changes in RBCs [37,45]. Many species of domestic and wild mammals show anemia and RBC morphological changes when infected by *T*. *evansi* [63–66]. Yet, despite the hematological alterations, RBC and PCV values were not much below values reported for captive uninfected animals [45,67]. A possible explanation for why most of the captured animals with high *T*. *evansi* parasitemias did not show very low RBC and PCV values is that although free-ranging coatis may develop anemia, severely affected individuals might not survive long in the wild. Alterations in RBC and PCV values can also be accompanied by a decrease in the number of platelets [68,69], as it was also found for coatis in this study. There is an array of possible reasons for a decrease in platelet numbers associated with *T*. *evansi* infection, including reduction of the platelet life span, splenic and hepatic sequestration, self-immune response and/or disseminated intravascular coagulation, characterized by systemic activation of blood coagulation, due to constant contact with trypomastigote forms and the vascular endothelium [12,69,70].

Changes in white blood cells due to infection by *T*. *evansi* in coatis have not been examined as closely as have changes in RBC and PCV [37,40]. A lower total WBCs in coatis with high parasitemias by *T*. *evansi* was previously detected [40], and was mainly caused by a lower number of neutrophils (including band cells) in males with high parasitemias of *T*. *evansi*. Species in the order Carnivora show varied responses to *T*. *evansi* infection regarding the number of circulating neutrophils [65,71,72], and reasons underpinning this variation are not known. Coatis also showed a reduced number of eosinophils, which may be a consequence of acute infection [12]. Waves of high parasitemia caused by variable surface glycoproteins are characteristic of this salivarian trypanosome and may be responsible for acute episodes during the course of infection. Eosinopenia (low number of eosinophils) was reported in cats experimentally infected with *T*. *evansi* [72], but the same was not observed in dogs [65,71]; indeed, interpretation of such results is troublesome, as eosinophil reference values for many species may be zero cells per mm^3^ of blood.

Finally, we observed a lower body condition of coatis with high parasitemias of *T*. *evansi* especially during the reproductive season. The lower body weight reflects a broader energetic challenge that is likely biologically meaningful for reproduction and survival of coatis, as it is for other species [6,8,73,74]. Trade-offs on resource allocation between host immune response and life history traits are expected and can play a significant role on host survival and/or reproduction [75], In this context, we speculate that adult coati males showing high *T*. *evansi* parasitemias during the reproductive season may be at a disadvantage when competing for access to groups of females.

### Possible effects of *Trypanosoma cruzi*

High parasitemias of *T*. *cruzi* were not associated with changes in RBC or PCV values of coatis, except perhaps for the three animals also infected with *T*. *evansi* (TC+TE+; Fig 1). However, total WBCs and neutrophils were slightly lower in coatis with high parasitemias of *T*. *cruzi* (Table 1 and Fig 2), whereas the decrease in the average number of monocytes was more pronounced. In mice, acute *T*. *cruzi* infection leads to alterations of the hematopoietic system, resulting in leukopenia amongst other alterations [76], but infection by *T*. *cruzi* has variable effects on host hematological parameters of different species [77–80]. Monocytes and lymphocytes, however, are believed to play an important role in halting *T*. *cruzi* during the early, acute stage of infection [79][81], with peripheral monocyte numbers increasing due to subacute and chronic inflammatory responses usually caused by bacterial and protozoan infections [12]. Therefore, the lower number of mature neutrophils and the decrease in monocyte numbers of animals with high parasitemias of *T*. *cruzi* suggest that coatis may not respond well to high parasitemias of *T*. *cruzi*. Nevertheless, some coatis kept high *T*. *cruzi* parasitemias for 7.5 months on average (N = 8) [40], which may mean that the effects of *T*. *cruzi* on coati health are more related to morbidity than mortality.

### Possible effects of other parasites

We do not know the microfilariae species occurring in coatis from the Pantanal and the impact of most of these filarids in wild species is unknown. Coatis are wild hosts for *Dirofilaria immitis* in Argentina [82] and *Brugia guyanensis* in French Guiana [83]; in Brazil, they are infected with *D*. *repens* and *D*. *incrassata* [84]. Infection by *D*. *immitis* cause moderate normocytic normochromic anemia in dogs by reducing RBC, hemoglobin concentration and PCV values [85][86]. We found a contribution of abundance of the microfilariae to reduced body condition, RBC and PCV values, as well as band neutrophil numbers in coatis. Given the high prevalence of microfilaria in adult coatis and the absence of animals with clinical signs of infection, we suggest this parasite by itself has a minor effect on coati health.

Overall, gastrointestinal parasites were not associated with hematological parameters or decreased body condition, even though other studies have found an association between the presence or abundance of such parasites and a decrease in host body condition [5,6,9,87]. However, it is important to consider that sample size for gastrointestinal analyses was small and the statistical power for these analyses is therefore low. Despite that, we found that intensity of cestode eggs was positively associated with number of eosinophils. Eosinophils are involved in the regulation of allergic and inflammatory responses, playing an active role in almost all types of inflammatory processes. Eosinophilia (increased number of circulating eosinophils) can be found as a response to several parasitic infections [12,14] and may occur as a response to cestode infections. The large size of helminths precludes them to be phagocytized and as a consequence, eosinophils release toxin molecules that combat helminths extracellularly. The abundance of coccidian oocysts was also slightly associated with increased numbers of circulating neutrophils. Coccidian infection can cause diverse alterations in the blood parameters of experimentally infected hosts [88,89] and their effect on coatis has never been investigated.

Identifying parasite community composition and relative species abundance from fecal samples is a challenge and not always feasible [11]. Even with the recent advances in molecular techniques for identification of helminth eggs and larvae [90][91], identification may be too costly or inefficient, especially if the host species is poorly known and the chances of finding new species of parasites is high. Despite such methodological drawbacks, analyzing aggregate-scale parasite data should not be discarded because strong physiological effects can still be detected [11] and the obtained results may guide future research on specific parasite groups. In this study, even though we had a small sample size for analyses involving gastrointestinal parasites and identification of helminths was not done at the species-level, we observed that parasites such as filarids, cestodes and coccidians are common in coatis from the Pantanal and that they may affect the health of coatis. Future research should therefore focus on the species-level identification of these species as well as on more refined methods of parasite quantification.

### Reproductive seasonality and sex differences

Overall, coati body condition was lower during the reproductive season, and the relation between high parasitemias by hemoparasites and changes in the hematological parameters was stronger during that season (Figs 1 and 2b). A reduction in body fat during the reproductive season has been observed for *N*. *narica* from Tikal National Park, Guatemala and probably reflects reproductive stress, perhaps associated with reduced food consumption [92]. During the reproductive season, poor nutrition and investment in reproduction weaken the immune system in ways that could affect the ability of hosts to defend against parasites [20–22].

Female coatis apparently cope better with infection by *T*. *evansi* than do males. The lower WBCs and neutrophils related to *T*. *evansi* detection were usually greater in males, especially during the reproductive season. Estrogen seems to play an important role in resistance to *T*. *cruzi* [93,94] and many other parasites, whereas testosterone depresses the immune system of males [23,25]. In addition, coatis are polygynous, show sexual dimorphism, and males strongly compete for females during the reproductive season, which sometimes result in wounds [51]. Given these behavioral and morphological characteristics, hematological differences between coati males and females are expected to be pronounced [95]. This would also contribute to an even stronger decrease in immune response during the mating season, when testosterone levels in coati males peak [96]. However, it is important to notice that sample sizes for positive *T*. *cruzi* or *T*. *evansi* females were small.

Finally, we observed that neutrophil numbers were higher mainly in males during the reproductive season. An increase in the number of circulating young neutrophils (shift to left) occurs when demand from the bone marrow increases and a large percentage of band cells are released in the bloodstream [12]. This finding may indicate that the challenge to the immune system of males is greater during that season, in agreement with the other findings described above.

### Concomitant infections

There is an increasing need for investigation of the concurrent impact of multiple parasites on host health [8,49,97]. The role of simultaneous infection by multiple parasites on host health is usually overlooked even though coinfection is the rule [49,98,99]. In this study we detected effects of concomitant infections on host health. For instance, filarids along with *T*. *evansi* were present in the best models explaining variation in body condition and RBC/PCV (Table 2). Likewise, WBC and neutrophils were lower in coatis infected with *T*. *cruzi* or *T*. *evansi*, and we found some evidence that such numbers may be even lower in double infected males (Fig 2a), a pattern suggested by Alves and co-workers [40].

The order of infection by multiple parasites and its effect on host health should be an important focus for future investigations [100], although it may be difficult to control for in free-ranging animals. In this context, experimental studies may be useful to disentangle the effect of each parasite on host health as well as to clarify the mechanisms by which parasites may affect host health. For instance, Rodriguez and co-workers [101] verified that the number of blood-circulating *T*. *cruzi* trypomastigotes in mice varied as a function of the development of early experimental infection with the cestode *Taenia crassiceps*, which would alter host susceptibility to *T*. *cruzi* infection and the magnitude of peaks in *T*. *cruzi* parasitemia. Therefore, patterns of high *T*. *cruzi* parasitemias in coatis may be at least partially influenced by co-infection with other parasites. Indeed, concurrent micro- and macroparasite infections may down-regulate different immunologic pathways. In general, the immune response to intracellular parasites involves T helper 1 (Th1) cells, while response against extracellular parasites are mediated by T helper 2 (Th2) cells. Cytokines produced by Th1 and Th2 cells suppress one another’s immune function [102–104]. Notwithstanding, field studies such as this work with coatis and *Trypanosoma* allow a more realistic approach when investigating the effect of parasites on host health in the wild. If a parasite has economic, veterinary or public health importance and the host is thought to be a reservoir, *in situ* studies become essential to understand and predict parasite persistence and impacts in reservoir populations.

## Conclusions

In this study, we observed that the health of coatis, as measured by hematological parameters along with body condition, was predicted by infection with parasites. We observed associations between high *Trypanosoma* and microfilariae parasitemias and differences on hematological parameters and body condition of free-ranging coatis. In addition, we found evidence of negative effects of co-infection, as well as sex and/or seasonality on coati health.

It is increasingly clear that ecologists need to address how diverse and co-occurring parasites affect wildlife health [11,97,100]. However, such broad approach is challenging, especially given a lack of knowledge regarding the parasitic fauna of most wild species and the difficulties of accurately diagnosing and quantifying infection by multiple parasites. These difficulties will only be overcome with the growth of multidisciplinary research collaborations and the understanding that the development of such an extensive approach may initially come at the expense of refined data.

## Supporting Information

S1 Table Hematological parameters for > 2 years old coatis. Mean (standard deviation); median; minimum ─ maximum value in the sample; N = sample size; N (Platelets) = sample size for platelet counts; PCV = packed cell volume; RBC = red blood cells count; WBC = total white blood cells counts. TC = *T*. *cruzi*; TE = *T*. *evansi*; (+) = high parasitemia; (-) = low parasitemia. The study was conducted in the Nhumirim Ranch, Pantanal from 2005 to 2009. High parasitemia by *T*. *evansi* was detected by the microhematocrit centrifuge technique (MHCT), whereas for *T*. *cruzi* it was detected by hemoculture (HC). Recaptures are not considered in this table.
(XLSX)
